# Genomic amplification upregulates estrogen-related receptor alpha and its depletion inhibits oral squamous cell carcinoma tumors *in vivo*

**DOI:** 10.1038/srep17621

**Published:** 2015-12-07

**Authors:** Ankana Tiwari, Shivananda Swamy, Kodaganur S. Gopinath, Arun Kumar

**Affiliations:** 1 Department of Molecular Reproduction, Development and Genetics, Indian Institute of Science, Bangalore 560012, India; 2 Department of Surgery, Bangalore Institute of Oncology, Bangalore 560027, India

## Abstract

The *ESRRA* gene encodes a transcription factor and regulates several genes, such as *WNT11* and *OPN*, involved in tumorigenesis. It is upregulated in several cancers, including OSCC. We have previously shown that the tumor suppressor miR-125a targets *ESRRA*, and its downregulation causes upregulation of ESRRA in OSCC. Upregulation of ESRRA in the absence of downregulation of miR-125a in a subset of OSCC samples suggests the involvement of an alternative mechanism. Using TaqMan^®^ copy number assay, here we report for the first time that the genomic amplification of *ESRRA* causes its upregulation in a subset of OSCC samples. Ectopic overexpression of ESRRA led to accelerated cell proliferation, anchorage-independent cell growth and invasion, and inhibited apoptosis. Whereas, knockdown of ESRRA expression by siRNA led to reduced cell proliferation, anchorage-independent cell growth and invasion, and accelerated apoptosis. Furthermore, the delivery of a synthetic biostable *ESRRA* siRNA to OSCC cells resulted in regression of xenografts in nude mice. Thus, the genomic amplification of *ESRRA* is another novel mechanism for its upregulation in OSCC. Based on our *in vitro* and *in vivo* experiments, we suggest that targeting *ESRRA* by siRNA could be a novel therapeutic strategy for OSCC and other cancers.

Oral squamous cell carcinoma (OSCC) or oral cancer is one of the most common malignancies world over, with an annual incidence of 300,000 cases^1^. In India, it is the leading cancer in males and the third most common cancer in females^2^. According to GLOBOCAN 2012 (http://globocan.iarc.fr), the incidence of OSCC in India is 69,820 cases annually. Despite recent advancements in treatment strategies including cetuximab, a monoclonal antibody against EGFR, the overall survival rate of OSCC patients has not significantly improved over the last few decades^2^. Hence, it is important to identify novel therapeutic targets for OSCC.

The *ESRRA* (estrogen-related receptor alpha) gene, located on chromosome 11q13.1, spans 13.5 Kb of genomic DNA with seven exons and codes for a 423-amino acid-long protein of 46 kDa. ESRRA harbors a DNA binding domain (amino acids 73–168), which is composed of two C4-type zinc fingers, a ligand binding domain (amino acids 197–420) and a monopartite nuclear localization signal (amino acids 71 LSSLPKRLCLV 81). It is expressed at high levels in tissues with a high energy requirement such as kidney, heart and skeletal muscles^3^. It functions as a transcription factor and positively regulates the transcription of *OPN* (osteopontin) and *WNT11* (wingless-type MMTV integration site family, member 11) genes involved in cell proliferation, migration and invasion^4^. The ESRRA protein exhibits a high degree of similarity with estrogen receptor alpha and binds to both estrogen response element (5′TCAAGGTCA3′) and its cognate response element (5′GGTCANNNTGACC3′)^5^.

Interestingly, ESRRA is shown to be upregulated in several cancers such as breast, prostate, ovarian, colon and oral cancers^6^,^7^,^8^,^9^,^10^,^11^. The homozygous deletion of *ESRRA* in a mouse model of ERBB2-induced mammary tumors causes a significant delay in tumor development^12^. Further, ESRRA is known to promote cancer cell migration and invasion^11^,^13^. Thus, the above observations implicate the role of ESRRA in tumorigenesis and also suggest that it could be an attractive target for anti-cancer therapy.

Given the role of ESRRA in tumorigenesis, not much is known about the mechanism underlying its upregulation in cancers. We have recently reported that the downregulation of tumor suppressor miR-125a is one of the major mechanisms for upregulation of ESRRA in a subset of OSCC samples^11^. In addition, we have also identified another subset of OSCC samples having an upregulated level of ESRRA in the absence of miR-125a downregulation, suggesting the involvement of some other mechanisms, such as genomic amplification, for its upregulation.

Using TaqMan^®^ copy number assay, here we show for the first time that the genomic amplification of *ESRRA* is another novel mechanism for its upregulation in OSCC. Using *in vitro* and *in vivo* experiments, we further show that targeting ESRRA via synthetic siRNAs could be a novel therapeutic strategy to treat OSCC and other cancers.

## Results

### Upregulation of ESRRA at mRNA and protein levels

**Figure 1:**
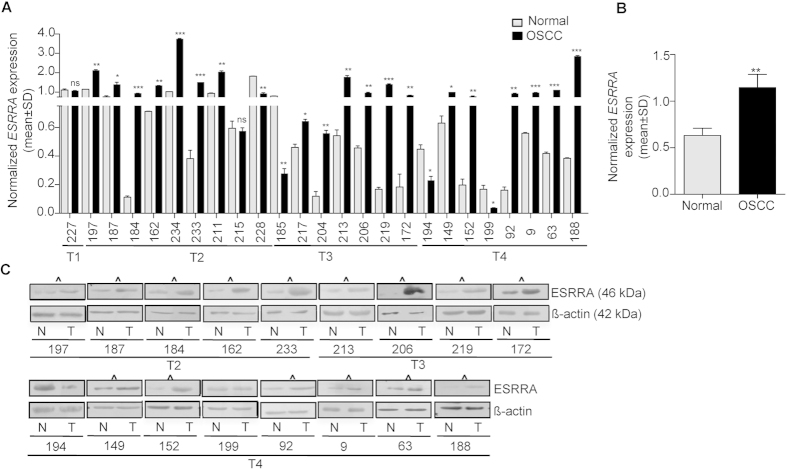
Expression of ESRRA in OSCC samples. (**A**) The qRT-PCR analysis of *ESRRA* in 25 matched normal oral tissue and OSCC samples. Numbers on the X-axis represent matched normal oral tissue and OSCC samples from different patients. T1, T2, T3 and T4 represent different grades of OSCC samples. (**B**) The comparison of combined qRT-PCR data of the *ESRRA* transcript level between normal oral tissue and OSCC samples. (**C**) The Western blot analysis of ESRRA in 17 matched normal oral tissue and OSCC samples. OSCC samples showing upregulation of ESRRA as compared to their matched normal oral tissues are marked by **^**. N and T represent normal oral tissue and OSCC samples respectively. β-actin was used as a loading control. *p < 0.05; **p < 0.01; ***p < 0.001; and ns, non-significant.

To assess if *ESRRA* is upregulated in OSCC, we used qRT-PCR to determine its transcript level in 25 matched normal oral tissue and OSCC samples. The results showed its upregulation in 19/25 (76%) OSCC samples (viz., #197, 187, 184, 162, 234, 233, 211, 217, 204, 213, 206, 219, 172, 149, 152, 92, 9, 63 and 188) and downregulation in 4/25 (16%) OSCC samples (viz., #228, 185, 194 and 199) as compared to their matched normal oral tissues (Fig. 1A). Moreover, 2/25 (8%) OSCC samples (viz., #227 and 215) did not show any significant change in its expression as compared to their matched normal oral tissue counterparts (Fig. 1A). When we compared the combined data from OSCC samples with that from the normal oral tissues, it was significantly upregulated in OSCC samples as compared to normal oral tissues (Fig. 1B).

To determine if there is a correlation between upregulation of ESRRA at the transcript and protein levels, we examined its expression at the protein level by Western blotting in 17/25 OSCC samples used previously in qRT-PCR analysis. We were not able to analyse 8/25 samples due to the unavailability of good quality protein lysates. The results showed that 15/17 (88.24%) OSCC samples (viz., #197, 187, 184, 162, 233, 213, 206, 219, 172, 149, 152, 92, 9, 63 and 188), which showed its upregulation at the mRNA level, also showed its upregulation at the protein level as compared to normal oral tissues (Fig. 1C). Of the remaining 2/17 samples, the OSCC sample #194 showed its downregulation at both the mRNA and protein levels (Fig. 1A,C). Whereas, the OSCC sample #199, which showed its downregulation at the mRNA level, showed no change at the protein level as compared to its matched normal oral tissue (Fig. 1A,C). Overall, the protein expression level of ESRRA corroborated well with its expression profile at the mRNA level in all but one case (sample #199).

### Genomic amplification of *ESRRA*

**Figure 2:**
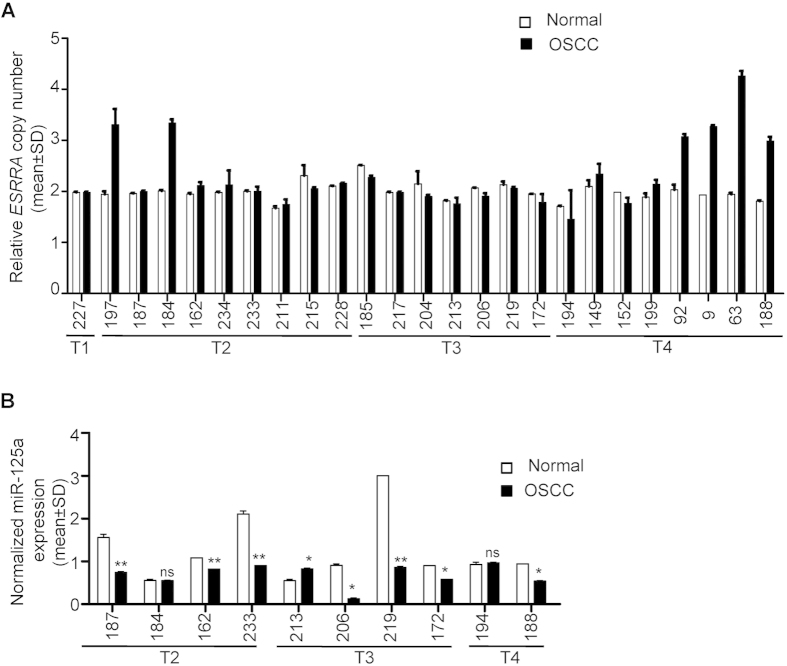
Genomic amplification of *ESRRA* and expression of miR-125a. (**A**) The TaqMan^®^ copy number assay to assess the genomic amplification of *ESRRA* in 25 matched normal peripheral blood and OSCC DNA samples. (**B**) The qRT-PCR analysis of miR-125a in 10 matched normal oral tissue and OSCC samples. *p < 0.05; **p < 0.01; and ns, non significant.

To determine if the upregulation of *ESRRA* in OSCC is due to its genomic amplification, we used TaqMan^®^ copy number assay to assess its amplification in 25 OSCC samples used above as compared to their matched peripheral blood DNA samples. The results showed its amplification in 6/25 (24%) OSCC samples (viz., #197, 184, 92, 9, 63 and 188) as compared to their matched normal blood DNA samples (Fig. 2A). The copy number of *ESRRA* ranged from three to four in these six OSCC samples (Fig. 2A). Interestingly, these six samples also showed its upregulation at the mRNA and protein levels (Figs 1A,C and 2A). Importantly, the proportion of individuals showing amplification of *ESRRA* in the OSCC samples (6/25, 24%) is statistically significantly higher than that in control (0/25, 0%) at the 95% confidence level by both Fisher’s exact test and binomial test (p < 0.05). The exact 95% confidence interval (based on binomial distribution) for the proportion showing amplification is from 9% to 45%, further demonstrating the adequacy of the sample size. These results suggested that the amplification of *ESRRA* is one of the mechanisms for its upregulation in OSCC.

As mentioned above, we had shown earlier that the downregulation of miR-125a is one of the mechanisms underlying upregulation of ESRRA in a subset of OSCC samples. Thus to explore the correlation between these two mechanisms, we analysed the expression of miR-125a in 10/25 OSCC samples (viz., #187, 184, 162, 233, 213, 206, 219, 172, 194 and 188) used in the present study (Fig. 2B). We could not analyse its expression in the remaining 15/25 samples due to unavailability of sufficient amounts of total RNA and tissue samples. A correlation was observed in 8/10 OSCC samples (viz., #187, 184, 162, 233, 206, 219, 172 and 188) between upregulation of ESRRA and its amplification (viz., OSCC sample #184 and 188) or the downregulated level of miR-125a (OSCC sample #187, 162, 233, 206, 219, 172 and 188). For example, the OSCC sample #184 showed an upregulation of *ESRRA* with a concomitant genomic amplification in the absence of miR-125a downregulation (Figs 1A,C and 2A,B and Supplementary Table S1). Whereas, the OSCC sample #162 showed upregulation of *ESRRA* with a concomitant downregulation of miR-125a in the absence of its genomic amplification (Figs 1A,C and 2A,B and Supplementary Table S1). Interestingly, both mechanisms were operational in the OSCC sample #188 as it showed upregulation of ESRRA with a concomitant genomic amplification as well as downregulation of miR-125a. Only in OSCC sample #194 and 213, no correlation was observed between expression of ESRRA and its amplification or the downregulated level of miR-125a (Figs 1A,C and 2A,B and Supplementary Table S1). For example, the downregulated level of *ESRRA* in OSCC sample #194 was accompanied by the absence of genomic amplification and no change in the level of miR-125a between normal oral tissue and OSCC (Figs 1A,Cand 2A,B and Supplementary Table S1). In OSCC sample #213, upregulation of *ESRRA* was accompanied by upregulation of miR-125a and the absence of genomic amplification (Figs 1A,C and 2A,B and Supplementary Table S1).

### Genomic amplification of *ESRRA* in OSCC cell lines

**Figure 3:**
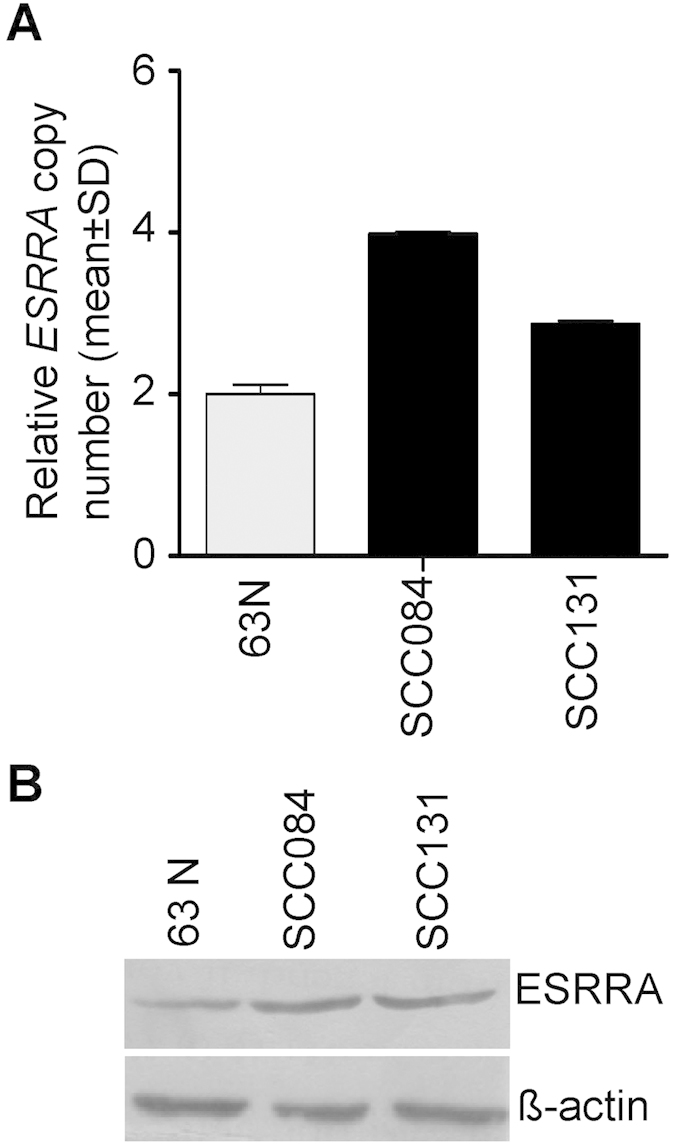
Genomic amplification and expression of ESRRA in OSCC cell lines. (**A**) The TaqMan^®^ copy number assay to assess the genomic amplification of *ESRRA* in OSCC cell lines. 63N represents peripheral blood DNA from patient #63. (**B**) The Western blot analysis to assess the level of ESRRA in OSCC cell lines. 63N represents normal oral tissue from patient #63.

In order to see if *ESRRA* is also amplified in OSCC cell lines, we used the Taqman^®^ copy number assay to assess its amplification status. As a control, we used the peripheral blood DNA sample from an OSCC patient #63. Interestingly, the *ESRRA* gene was amplified in both the OSCC cell lines (Fig. 3A). Further, SCC084 and SCC131 cell lines harboured four and three copies of *ESRRA*, respectively (Fig. 3A). To determine if the amplification of *ESSRA* causes its upregulation at the protein level, we used Western blot analysis. As expected, the level of ESRRA protein was increased in both cell lines as compared to normal oral tissue from patient #63 (Fig. 3B).

### Effect of overexpression of ESRRA

**Figure 4:**
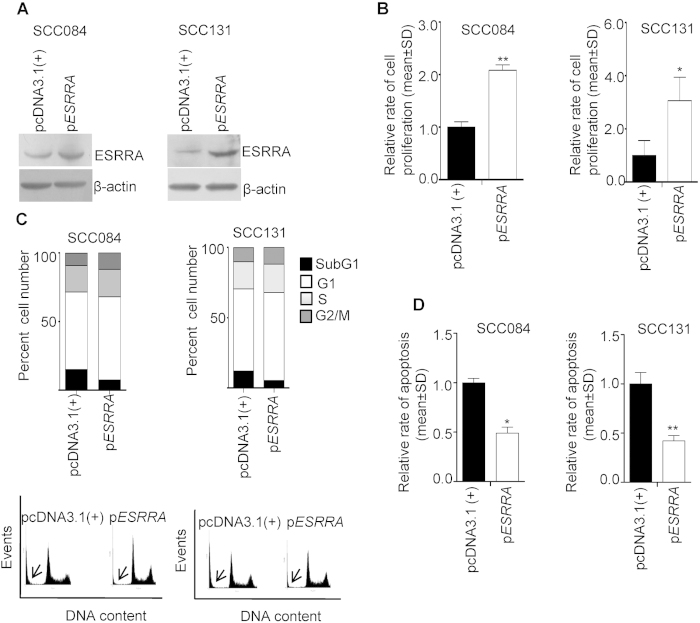
The effect of ESRRA overexpression on the proliferation and apoptosis of OSCC cells. (**A**) The Western blot analysis to assess the levels of ESRRA following transfection of cells with p*ESRRA.* (**B**) The effect of ESRRA overexpression on the proliferation of cells by the BrdU assay. (**C**) The FACS analysis of PI-stained cells transfected with pcDNA3.1(+) and p*ESRRA*. A graphical representation of the data is shown below. The subG1 population is marked by an arrow. (**D**) The effect of ESRRA overexpression on the rate of apoptosis. *p < 0.05; and **p < 0.01.

As mentioned above, ESRRA is known to regulate cancer cell proliferation. To this end, we overexpressed ESRRA in OSCC cell lines SCC084 and SCC131 by transfecting a full-length *ESRRA* construct (p*ESRRA*). The overexpression of ESRRA was confirmed by Western blot analysis (Fig. 4A). As expected, the cells transfected with p*ESRRA* showed an increased level of ESRRA as compared to cells transfected with the vector control pcDNA3.1(+) (Fig. 4A). We further analysed the functional relevance of an increased level of ESRRA on the proliferation of SCC084 and SCC131 cells by the BrdU assay. The results showed that the ectopic overexpression of ESRRA results in a significantly increased rate of cell proliferation as compared to the vector transfected cells (Fig. 4B).

To determine the changes in cell cycle phases, we stained ESRRA overexpressing SCC084 and SCC131 cells with PI (propidium iodide) and subjected them to flow cytometry. The results showed a lesser accumulation of p*ESRRA* transfected cells in subG1 (an indication of cell death) as compared to pcDNA3.1(+) transfected cells (Fig. 4C). To assess the type of cell death, we quantitated the rate of apoptosis in ESRRA overexpressing SCC084 and SCC131 cells by the caspase-3 activation assay. The results demonstrated that the cells transfected with p*ESRRA* showed a significantly decreased rate of apoptosis as compared to pcDNA3.1(+) transfected cells (Fig. 4D).

### Effect of knock down of ESRRA

To see the effect of ESRRA knock down on cell proliferation, we first tested the specificity of *ESRRA* siRNA by transfecting SCC084 cells with *ESRRA* siRNA and then assessing the level of *ESRRA* transcript by qRT-PCR. As a control, we also transfected *WT1* siRNA and assessed its effect on the level of *ESRRA*. As shown in Supplementary Fig. S1, transfection of *ESRRA* siRNA significantly reduced its level. However, the levels of irrelevant genes, *WT1* and *STIL*, remained unchanged, suggesting the specificity of *ESRRA* siRNA (Supplementary Fig. S1). Further, the knock down of *WT1* by siRNA reduced its level, without changing the levels of *ESRRA* and *STIL* (Supplementary Fig. S1).

**Figure 5:**
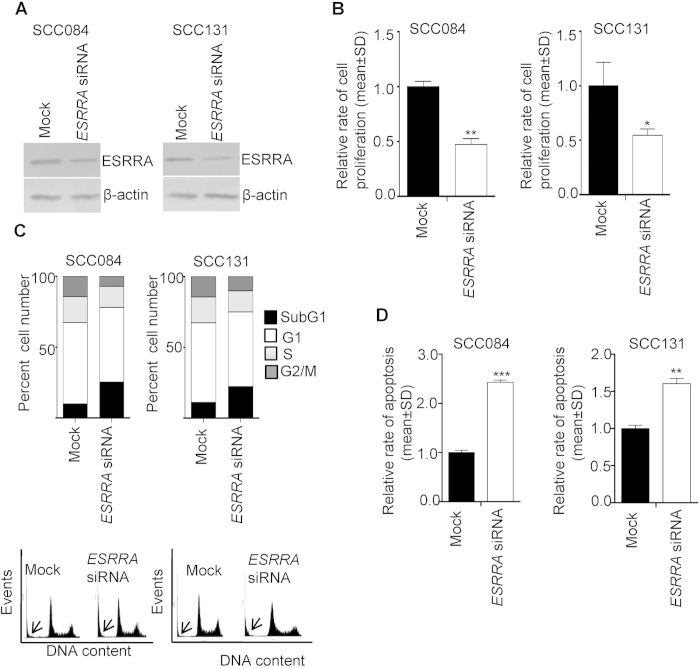
The effect of ESRRA knock down on the proliferation and apoptosis of OSCC cells. (**A**) The Western blot analysis to assess the level of ESRRA following transfection of cells with *ESRRA* siRNA. (**B**) The effect of ESRRA knock down on the proliferation of cells by the BrdU assay. (**C**) The FACS analysis of PI-stained cells transfected with mock and *ESRRA* siRNA. A graphical representation of the data is shown below. The subG1 population is marked by an arrow. (**D**) The effect of ESRRA knock down on the rate of apoptosis. *p < 0.05; **p < 0.01; and ***p < 0.001.

We further tested the effect of *ESRRA* specific siRNA on its protein level by Western blot analysis (Fig. 5A). As expected, OSCC cells transfected with *ESRRA* siRNA showed a reduced level of ESRRA as compared to mock (scrambled oligos) transfected cells (Fig. 5A). We then analysed the functional relevance of a decreased level of ESRRA on the proliferation of SCC084 and SCC131 cells by the BrdU assay. As shown in Fig. 5B, *ESRRA* siRNA transfected cells showed a significantly reduced rate of cell proliferation as compared to mock transfected cells.

To determine the changes in cell cycle phases, we stained the ESRRA knock down SCC084 and SCC131 cells with PI and subjected them to flow cytometry. The results showed a higher accumulation of *ESRRA* siRNA transfected cells in subG1 as compared to mock transfected cells (Fig. 5C). To assess the type of cell death, we quantitated the rate of apoptosis in ESRRA knock down SCC084 and SCC131 cells by the caspase-3 activation assay. The results demonstrated that the cells transfected with *ESRRA* siRNA showed a significantly increased rate of apoptosis as compared to mock transfected cells (Fig. 5D). The above observations suggested that ESRRA positively regulates the rate of cell proliferation in both the cell lines by decreasing the rate of apoptosis.

### Effect of ESRRA overexpression and knock down on anchorage-independent cell growth and invasion

**Figure 6:**
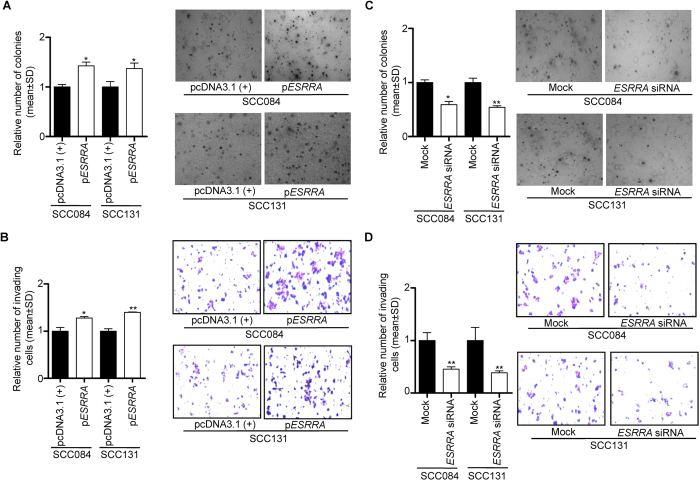
The effect of ESRRA overexpression and knock down on soft agar colony formation and cell invasion of OSCC cells. (**A**) The effect of ESRRA overexpression on the anchorage-independent growth of OSCC cells by the soft agar assay. (**B**) The effect of ESRRA overexpression on the invasion of OSCC cells by the transwell invasion assay. (**C**) The effect of ESRRA knock down on the anchorage-independent growth of OSCC cells by the soft agar assay. (**D**) The effect of ESRRA knock down on the invasion of OSCC cells by the transwell invasion assay. Microphotographs of soft agar colony formation and transwell invasion assays are also shown. *p < 0.05; and **p < 0.01.

To see the effect of overexpression and knock down of ESRRA on anchorage-independent cell growth and invasion, we overexpressed and knock down it in OSCC cells (viz., SCC084 and SCC131) by transfecting the p*ESRRA* construct and *ESRRA* siRNA, respectively. The colony formation and invasion potential of OSCC cells were assessed by soft agar and transwell migration assays, respectively. The cells transfected with p*ESRRA* showed an increased number of colony formation (Fig. 6A) and invaded cells in the transwell assay (Fig. 6B) as compared to cells transfected with the vector control, whereas the cells transfected with *ESRRA* siRNA showed lesser colony formation (Fig. 6C) and invaded cells in the transwell assay (Fig. 6D) as compared to mock transfected cells. These results suggested that ESRRA regulates anchorage-independent growth and invasive ability of OSCC cells.

### Effect of ESRRA overexpression and knock down on its transcriptional targets

**Figure 7:**
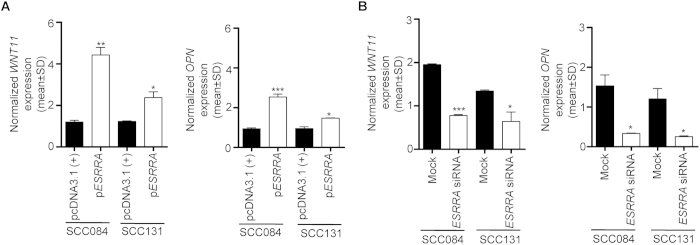
The effect of ESRRA overexpression and knock down on its transcriptional targets. (**A**) The qRT-PCR analysis to assess the level of *WNT11* and *OPN* transcripts in cells transfected with pcDNA3.1(+) and p*ESRRA*. (**B**) The qRT-PCR analysis to assess the level of *WNT11* and *OPN* transcripts in cells transfected with mock and *ESRRA* siRNA. *p < 0.05; **p < 0.01; and ***p < 0.001.

As stated above, ESRRA is a well known transcription factor and is known to transcriptionally regulate *WNT11* and *OPN*. To see the effect of overexpression and knock down of ESRRA on its transcriptional targets, we transfected the p*ESRRA* construct and *ESRRA* siRNA and quantitated the levels of *WNT11* and *OPN* by qRT-PCR (Fig. 7). The levels of *WNT11* and *OPN* were significantly increased in p*ESRRA* transfected cells as compared to vector transfected cells (Fig. 7A). Whereas, the levels of *WNT11* and *OPN* were significantly reduced in *ESRRA* siRNA transfected cells as compared to mock transfected cells (Fig. 7B).

### Depletion of ESRRA suppresses tumor growth *in vivo*

**Figure 8:**
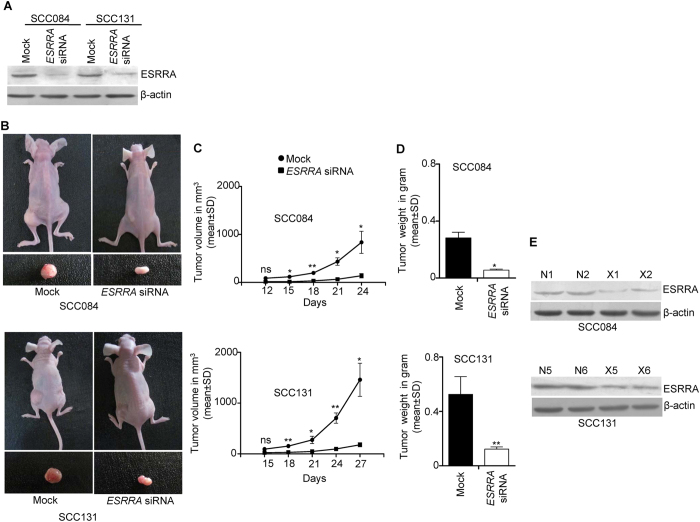
Depletion of ESRRA suppresses tumorigenicity *in vivo*. (**A**) The Western blot analysis to assess the level of ESRRA following transfection of SCC084 and SCC131 cells with mock and *ESRRA* siRNA. (**B**) The effect of mock and *ESRRA* siRNA on SCC084 and SCC131 cells derived xenografts in nude mice. Top panel: photographs of nude mice showing tumor growth after injection. Bottom panel: excised xenografts. (**C**) The effect of mock and *ESRRA* siRNA on the volume of SCC084 and SCC131 cells derived xenografts. (**D**) The effect of mock and *ESRRA* siRNA on the weight of SCC084 and SCC131 cells derived xenografts. (**E**) The Western blot analysis to assess the level of ESRRA in SCC084 and SCC131 cells derived xenografts. N1, N2, N5 and N6 represent xenografts with mock.X1, X2, X5 and X6 represent OSCC cells derived xenografts with *ESRRA* siRNA. Abbreviations: mock, Stealth RNAi^TM^ mock; and *ESRRA* siRNA, Stealth RNAi^TM^ *ESRRA* siRNA. *p < 0.05; **p < 0.01; and ns, non-significant. Four nude mice were used for each transfection.

Based on the results of *in vitro* studies, we hypothesized that the depletion of ESRRA in OSCC cells might have an anti-tumor effect *in vivo*. To address this hypothesis, we used an *in vivo* pretreated xenograft nude mouse model. We first tested the specificity of a biostable Stealth RNAi^TM^ *ESRRA* siRNA by transfecting it in SCC084 cells and then assessing the level of *ESRRA* transcript by qRT-PCR. As shown in Supplementary Fig. S1, ESRRA knock down significantly reduced its level. However, the levels of irrelevant genes, *WT1* and *STIL*, remained unchanged, suggesting the specificity of Stealth RNAi^TM^ *ESRRA* siRNA (Supplementary Fig. S1). We then tested the effect of this *ESRRA* specific Stealth RNAi^TM^ siRNA on its level by Western blot analysis (Fig. 8A). OSCC cells transfected with Stealth RNAi^TM^ *ESRRA* siRNA showed a reduced level of ESRRA as compared to Stealth RNAi^TM^ mock transfected cells in both cell lines (Fig. 8A). Post 24 hours of transfection of Stealth RNAi^TM^ *ESRRA* siRNA in SCC084 and SCC131 cells, these cells were injected separately into nude mice. The mice were observed for the growth of tumors till 24 days for SCC084 and 27 days for SCC131 pretreated cells. As shown in Fig. 8B–D, pre-transfection of Stealth RNAi^TM^ *ESRRA* siRNA into OSCC cells led to a significant reduction in both tumor volume and weight as compared to mock transfectants. Further, we also assessed the level of ESRRA protein in xenografts by Western blotting. The level of ESRRA was reduced in xenografts with Stealth RNAi^TM^ *ESRRA* siRNAas compared to those with Stealth RNAi^TM^ mock (Fig. 8E).

## Discussion

Upregulation of ESRRA has been documented in various cancer cell lines and breast, colorectal, prostate gland, ovarian, cervical and oral cancers^6^,^7^,^8^,^9^,^10^,^11^, underlying its potential role in progression of multiple cancers. Of note, its upregulation has been considered to be a negative prognostic factor in breast, ovarian and prostate tumors^6^,^7^,^8^. In the case of ovarian cancer, the expression level of ESRRA is known to serve as a biomarker for the disease, as its expression profile correlates with serum CA-125 levels^9^. In the present study, we have also observed upregulation of ESRRA at both the mRNA and protein levels in a majority of the OSCC samples (Fig. 1A,C).

Genomic amplification is one of the mechanisms for overexpression of oncogenes. Santarius *et al.*^14^ and Matsui *et al.*^15^ have performed a census of amplified genes across different tumors and have listed 81 genes. Using TaqMan^®^ copy number assay, we have shown for the first time that the *ESRRA* gene is amplified in 6/25 (24%) OSCC samples (Fig. 2A). The concomitant upregulation of ESRRA at both the mRNA and protein levels and its genomic amplification in a subset of OSCC samples suggested that the genomic amplification is one of the mechanisms for its upregulation. The present study thus increases the spectrum of amplified genes in cancers to 82.

*ESRRA* is located at chromosome band 11q13.1, which is shown to be amplified in breast, lung, liver, bladder, oesophageal and oral cancers^16^. Huang *et al.*^16^ have delineated the amplified region between the proximal marker D11S4178 (chromosome position 68189101 bp) and the distal marker D11S1314 (chromosome position 72323144 bp). This region harbours several genes, including *CCND1*, *FGF3*, *FGF4, TAOS1* and *EMS1* oncogenes. However, *ESRRA* is located outside the proximal boundary of this amplified region between chromosome positions 64073044 to 64084210 bp (UCSC Genome Bioinformatics site; http://genome.ucsc.edu/).

Intriguingly, we have observed several OSCC samples with an upregulated level of ESRRA in the absence of its genomic amplification, suggesting the involvement of an alternative mechanism for its upregulation in these samples. Since, we had shown previously that the downregulation of miR-125a causes upregulation of ESRRA^11^, we assessed the level of miR-125a in some samples which showed upregulation of ESRRA. The results showed that, in some OSCC samples (viz., #187, 162, 233, 206, 219 and 172), the upregulation of ESRRA was due to downregulation of miR-125a. Interestingly, in the OSCC sample #188, both the mechanisms seem to operate (Figs 1A,C and 2A,B).

Although we have observed a positive correlation between mRNA and protein levels of ESRRA in a majority of samples, there is discordance in some samples between mRNA and protein levels. For example, the OSCC sample #199 showed discrepancy in the levels of mRNA and protein (Fig. 1A,C). This sample showed downregulation of ESRRA at the mRNA level, but no change at the protein level. We speculate that this could be due to intratumoral tissue heterogeneity^17^.

The occurrence and development of cancer often involve two aspects, excessive proliferation followed by inhibited apoptosis. Previously, ESRRA has been shown to promote tumor cell proliferation^11^. In the present study, this observation was further confirmed by ectopic overexpression of ESRRA, which led to accelerated cell proliferation and inhibited apoptosis as compared to vector control cells (Fig. 4B–D). Whereas, the knock down of ESRRA expression by siRNA led to a reversed phenomenon; the ESRRA knock down cells exhibited reduced cell proliferation (Fig. 5B) and accelerated apoptosis (Fig. 5D). In concordance with our results, Zhao *et al.*^13^ have also observed previously that the siRNA knock down of *ESRRA* impaired the proliferation of breast cancer cell lines. Bernatchez *et al.*^18^ have shown that the lentivirus-based shRNA knock down of ESRRA impaired cellular growth of colon cancer cells from HCT116, DLD1 and HT29 cell lines. It has recently been reported that the overexpression of ESRRA promotes *in vitro* cell proliferation and malignant growth capacities of cells from prostate cancer cell lines LNCaP and PC-3 under both normal and hypoxic conditions^19^. Zou *et al.*^19^ have further demonstrated that the knock down of ESRRA in LNCaP cells leads to a significant decrease in cell proliferation under both normal and hypoxic conditions.

We have shown that the overexpression of ESRRA in OSCC cells promotes anchorage-independent cell growth and invasion, whereas its knock down decreases anchorage-independent cell growth and invasion (Fig. 6). These results are in concordance with our previous report^11^ and other reports^13^,^18^,^19^,^20^, suggesting the crucial role of ESRRA in tumorigenesis.

ESRRA is shown to transcriptionally regulate several genes, such as *WNT11* and *OPN*, which are its important effectors and implicated in cancer cell proliferation, migration and invasion^4^,^21^,^22^,^23^. Interestingly, *OPN* has been reported to regulate the proliferation of OSCC cells^24^. To explore the mechanism underlying the ESRRA mediated regulation of cancer cell proliferation and invasion, we analysed the levels of ESRRA target genes *WNT11* and *OPN* by overexpressing and knocking down its expression (Fig. 7). Our results showed that perturbing the level of ESRRA leads to alteration in the levels of *WNT11* and *OPN* (Fig. 7). These results suggest that overexpression of ESRRA in OSCC cells results in increased levels of *WNT11* and *OPN*, which in turn regulate OSCC cell proliferation and invasion.

Upregulation of ESRRA in several cancers, including OSCC, suggests that it could be used as a therapeutic target. Previously, it has been shown that the pretreatment of breast cancer cells with an inverse agonist of ESRRA, XCT790, can delay *in vivo* tumour growth in mice^25^. Further, Bernatchez *et al.*^18^ have shown that the lentivirus-based shRNA mediated silencing of *ESRRA* significantly reduces *in vivo* tumor growth of colon cancer cell line HCT116 derived xenografts. We therefore sought to explore if the knock down of ESRRA by a synthetic biostable siRNA could confer anti-tumor effect *in vivo* in OSCC cells. The results of our nude mice xenograft study with a biostable synthetic *ESRRA* siRNA suggest that it could be a potential therapeutic target in OSCC (Fig. 8).

In summary, we have shown for the first time that the genomic amplification of *ESRRA* is another mechanism for its upregulation in OSCC. Based on our *in vitro* and *in vivo* studies, we suggest that depleting the level of ESRRA via siRNA could be a key therapeutic strategy to treat OSCC and other cancers.

## Materials and Methods

### Clinical material

A total of 25 matched normal oral tissues (approximately 5 cm from the lesion) and OSCC samples were collected at the Bangalore Institute of Oncology, Bangalore. All experiments were carried out in accordance with the approved guidelines. Tissue samples were collected following informed consent from the patients and approval from the ethics committee of the Bangalore Institute of Oncology. Tissue samples were collected in the RNAlater™ (Sigma-Aldrich, St. Louis, MO) immediately after surgery or biopsy. Peripheral blood samples from patients were also collected in EDTA-Vacutainer^TM^ blood collection tubes (Becton-Dickinson, Franklin Lakes, NJ). Patients enrolled in the study were not under any treatment at the time of the surgery. Tumors were classified according to the TNM (Tumor, Node and Metastasis) classification based on the UICC (Union for International Cancer Control, Switzerland; http://www.uicc.org/resources/how-use-tnm-classification). Details of patients are given in Supplementary Table S1.

### Total RNA isolation

Total RNA was isolated from tissues and cell lines, using the TRI Reagent^®^ (Sigma-Aldrich, St. Louis, MO).

### qRT-PCR analysis

For gene expression analysis, the first-strand cDNA was synthesized using a RevertAid™ H Minus First Strand cDNA Synthesis kit (MBI Fermentas, Burlington, Canada), random hexamer primers and 1.5 μg RNA, as suggested by the manufacturer.

The quantitative real-time RT-PCR (qRT-PCR) was carried out using the Dynamo SYBR Green Mix (Finzymes, Espoo, Finland) in an ABI Prism^®^ 7900HT Sequence Detection System (Applied Biosystems, Foster City, CA), gene specific primers and *GAPDH* as a normalizing control. Normalized expression of a gene (viz., *ESRRA*, *WT1*, *STIL*, *WNT11* and *OPN*) was calculated as follows: ΔC(t) gene = C(t) gene-C(t) *GAPDH*, where C(t) is the cycle threshold value, and ΔC(t) represents the gene expression normalized to *GAPDH*. The student’s t-test was used to determine the significance of the difference in gene expression, using GraphPad PRISM5 software (GraphPad Software Inc., San Diego, CA). Details of the qRT-PCR primers are given in Supplementary Table S2.

### Genomic amplification

Genomic amplification of *ESRRA* was determined using the TaqMan^®^ copy number assay (Life Technologies, Grand Island, NY), which is a sensitive technique and has been extensively used to assess copy number variation of various genes in tumor DNA samples as compared to normal DNA samples from peripheral blood^26^,^27^,^28^,^29^,^30^,^31^,^32^. Genomic DNA from peripheral blood and OSCC samples was isolated using a Wizard^®^ Genomic DNA Purification kit (Promega, Madison, WI). Quantitative PCR (qPCR) was performed in an ABI Prism^®^ 7900HT Sequence Detection System, using an *ESRRA* specific TaqMan^®^ copy number assay kit. Experiments were carried out using 20 ng of genomic DNA as a template for each sample. The *RNase P* (*RPPH1*) gene was co-amplified with *ESRRA* and used as a standard reference gene for normalization (Life Technologies, Grand Island, NY). The assay involves relative quantification of the *ESRRA* sequence versus the *RNase P* sequence known to have two copies per diploid genome. Normalized values of *ESRRA* amplification in OSCC and peripheral blood (normal) DNA were calculated as follows: ΔC(t) OSCC = C(t) *ESRRA*-C(t) *RNase P*, and ΔC(t) Normal = C(t) *ESRRA*-C(t) *RNase P*, where Ct is the cycle threshold value, and ΔCt represents the *ESRRA* value normalized to *RNase P*. The relative copy number is plotted as 2 * 2^(−ΔC(t))^29^.

### qRT-PCR analysis of miR-125a

The qRT-PCR analysis of miR-125a was carried out by miR-Q, a method developed by Sharbati-Tehrani *et al.*^32^ for the expression analysis of miRNAs in an ABI Prism^®^ 7900HT sequence detection system, using the Dynamo SYBR Green Mix, RT6 miR-125a primer and a Revert Aid™ H Minus First Strand cDNA Synthesis kit. *5S RNA* was used as a normalizing control.

The miR-125a expression normalized to *5S RNA* was calculated using the following equation: ΔC(t) miR-125a = C(t) miR-125a-C(t) *5S RNA*, where C(t) is the cycle threshold value, and ΔC(t) represents the miR-125a expression normalized to *5S RNA*. The statistical significance of the difference in miR-125a expression between normal oral tissue and OSCC samples was determined by two-tailed unpaired t-test, using the GraphPad PRISM5 software. Details of the qRT-PCR primers are listed in Supplementary Table S2.

### Cell culture

Following two human oral squamous cell carcinoma cell lines were used in this study: SCC084 and SCC131. These cell lines were a kind gift from Prof. Susanne M. Gollin, University of Pittsburgh, Pittsburgh, PA. Cells were grown in Dulbecco’s modified Eagle’s Medium (DMEM) supplemented with 10% fetal bovine serum (Sigma-Aldrich, St. Louis, MO) at 37 °C in 5% CO_2_.

### Western blot analysis

Preparation of total protein lysates from tissues and cell lines, and Western blot analysis were carried out as described in Tiwari *et al.*^11^. Since the anti-goat ESRRA antibody (cat#sc-32790; Santa Cruz Biotechnology, Santa Cruz, CA) used in our study was not suitable for immuno-histochemistry, we analysed the expression of ESRRA in tissue samples using Western hybridization. To ensure equal protein loading in gels, an anti-mouse β-actin antibody (cat#A5441; Sigma-Aldrich, St. Louis, MO) was used. Signals on the membrane were detected as described in Tiwari *et al.*^11^.

### siRNA and plasmid construct

For *in vitro* experiments, mock siRNA (scrambled) and *ESRRA* specific siRNA were purchased from Life Technologies (Grand Island, NY). The *WT1* specific siRNA was purchased from Dharmacon Thermo Fisher Scientific (Pittsburgh, PA). Transient transfection of *ESRRA* siRNA (100 nM), *WT1* siRNA (100 nM) and mock (100 nM) was performed using Lipofectamine 2000 (Life Technologies, Grand Island, NY). For *in vivo* experiments, biostable Stealth RNAi^TM^ mock (scrambled) siRNA and Stealth RNAi^TM^ *ESRRA* specific siRNA were purchased from Life Technologies, and transfected in cells using Lipofectamine 2000. The p*ESRRA* construct harboring a full-length *ESRRA* (GeneBank accession no. NM_001282450.1) ORF was generated in the pcDNA3.1(+) vector by PCR using gene specific primers (Supplementary Table S3) and cDNA template from a normal oral tissue.

### Cell proliferation assay

The rate of cell proliferation was calculated using a CHEMICON® BrdU Cell Proliferation Assay Kit (Milipore Corporation, Billerica, MA) as described previously^11^. The relative rate of cell proliferation was calculated according to Jiang *et al.*^33^.

### Cell cycle analysis and detection of caspase-3 activation

For the cell cycle analysis, cells were stained with propidium iodide (Sigma-Aldrich, St. Louis, MO) and analysed by a FACScalibur™ flow cytometer (BD Biosciences, San Jose, CA), as described previously^11^. Cells were treated with RNase to ensure that PI staining is DNA specific^11^. The caspase-3 activation was determined using a caspGLOW™ Fluorescein Active Caspase-3 Staining kit (Biovision, Mountain View, CA) as suggested by the manufacturer. The relative rate of apoptosis was calculated according to Jiang *et al.*^33^.

### Soft agar colony forming assay

The soft agar colony forming assay was performed using 2,500 cells for each plate, as described previously^11^,^34^. Briefly, 24 hr post transfection with mock or *ESRRA*-specific siRNA, cells were mixed with DMEM containing soft agar (Difco, Mubai, India) to a final concentration of 0.35%. These cells were then plated in a 35 mm dish coated with 0.6% soft agar in DMEM. At the end of 10 days, the number of colonies in three random microscopic fields were counted, and the images were captured using an Olympus CKX41 phase contrast microscope (Olympus Optical Co., Tokyo, Japan).

### Cell invasion assay

The cell invasion assay was performed using a BD BioCoat™ Matrigel™ Invasion Chamber (BD Biosciences, San Jose, CA), as described in Tiwari *et al.*^11^. Cells were photographed and counted in three random microscopic fields under a 10× objective to calculate the number of cells that had invaded through the matrigel matrix. The graph was plotted for the number of cells that had invaded per microscopic field.

### *In vivo* assay for tumor growth

To see the effect of ESRRA depletion on tumor growth, 2 × 10^6^ SCC084 or SCC131 cells were transfected separately with synthetic biostable Stealth RNAi^TM^ *ESRRA* specific siRNA (200 nM) or Stealth RNAi^TM^ mock (200 nM). Post 24 hr of transfection, cells from each transfection were suspended separately in 150 μl of incomplete DMEM and then subcutaneously injected into the right flank of a female BALB/c athymic 6-week old nude mouse. Four nude mice were used in each transfection experiment. Tumors (xenografts) of measurable size appeared post 12 days of injection. Tumor growth was monitored, and its volume was measured using a digital calliper. Tumor volume (V) was calculated as follows: V = L × W^2^ × 0.5, where L and W represent length and width respectively. All nude mice experiments were performed in accordance with the institute guidelines and approved by the animal ethics committee of the Indian Institute of Science.

## Additional Information

**How to cite this article**: Tiwari, A. *et al.* Genomic amplification upregulates estrogen-related receptor alpha and its depletion inhibits oral squamous cell carcinoma tumors *in vivo*. *Sci. Rep.* **5**, 17621; doi: 10.1038/srep17621 (2015).

## Supplementary Material

Supplementary Information
