# Author Correction: Genomic amplification upregulates estrogen-related receptor alpha and its depletion inhibits oral squamous cell carcinoma tumors *in vivo*

**DOI:** 10.1038/s41598-018-35662-3

**Published:** 2018-11-19

**Authors:** Ankana Tiwari, Shivananda Swamy, Kodaganur S. Gopinath, Arun Kumar

**Affiliations:** 1 Department of Molecular Reproduction, Development and Genetics, Indian Institute of Science, Bangalore, 560012 India; 2 Department of Surgery, Bangalore Institute of Oncology, Bangalore, 560027 India

Correction to: *Scientific Reports* 10.1038/srep17621, published online 07 December 2015

This Article contains an error in Figure 6A, where an incorrect p*ESRRA* microphotograph for SCC084 was inadvertently included. The correct Figure 6A is given below as Figure 1.

**Figure 1:**
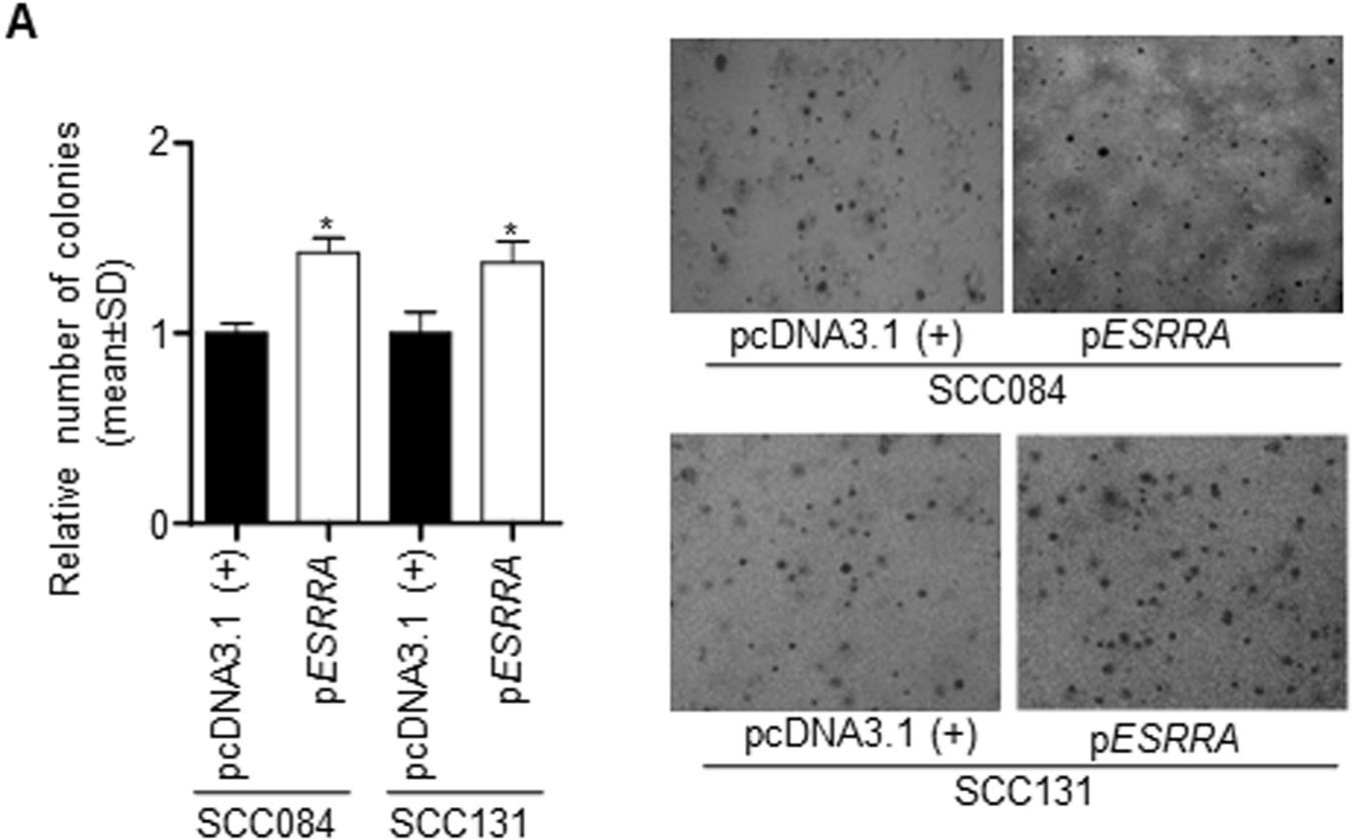
The effect of ESRRA overexpression and knock down on soft agar colony formation and cell invasion of OSCC cells. (**A**) The effect of ESRRA overexpression on the anchorage-independent growth of OSCC cells by the soft agar assay.

