# Supplementary material for: Genomic amplification upregulates estrogen-related receptor alpha and its depletion inhibits oral squamous cell carcinoma tumors in vivo
Source: Sci Rep. 2015 Dec 7;5:17621. doi: 10.1038/srep17621 (PMC4671026; doi:10.1038/srep17621)
Supplement: Supplementary Information [file srep17621-s1.pdf]

Supplementary information

Genomic amplification upregulates estrogen-related receptor alpha and its depletion inhibits oral squamous cell carcinoma tumors *in vivo*

Ankana Tiwari, Shivananda Swamy, Kodaganur S. Gopinath and Arun Kumar

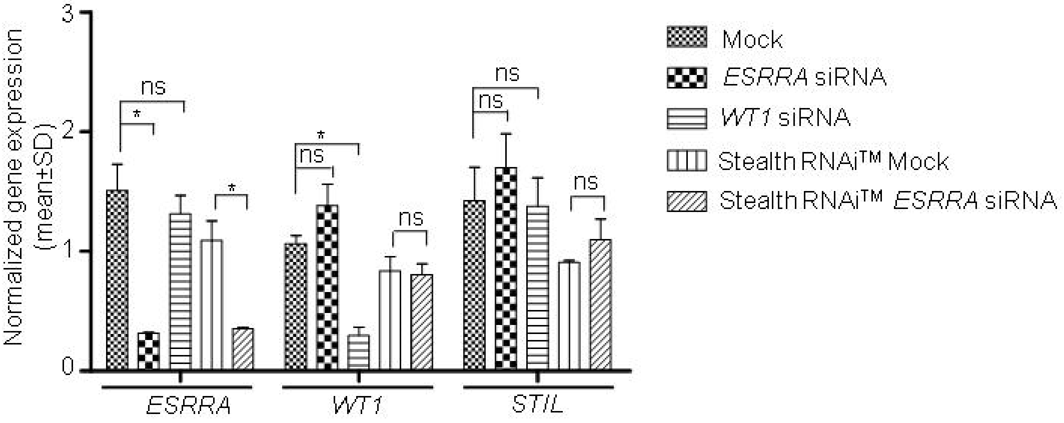

Supplementary Figure S1. The qRT-PCR analysis of *ESRRA*, *WT1* and *STIL* expression in SCC084 cells transfected with siRNAs targeting *ESRRA* and *WT1*. Note, SCC084 cells transfected with *ESRRA* siRNA show a significant knock down of *ESRRA*. siRNA targeting *WT1* was used as a control. *, $p<0.05$; and ns, non-significant.

Supplementary Table S1. Details of clinicopathological features of patients and molecular data.

**Sl.#**	**Pt (sample) #**	**Age (yr)/Sex**	**Site of the tumor**	**TNM**	**Tob/Alco/ Bet/Smok usage**	***ESRRA*** **(amplification)**	***ESRRA*** **(mRNA)**	**ESRRA (Protein)**
1	9	45M	Tongue-anterior 2/3	T4N2M0	Chronic Tob chewing	Yes	Up	Up
2	63	40F	BM	T4aN1MX	Chronic Tob chewing	Yes	Up	Up
3	92	50F	BM	T4N2bM0	Chronic Tob chewing	Yes	Up	Up
4	149	50M	BM	T4N2cMx	Tob chewing	No	Up	Up
5	152	65F	BM	T4N1M0	Tob chewing	No	Up	Up
6	162	55F	BM	T2N0M0	Chronic Tob chewing	No	Up	Up
7	172	57F	BM	T3N0M0	Chronic Bet chewing	No	Up	Up
8	184	38M	Tongue-anterior 2/3	T2N0M0	No	Yes	Up	Up
9	185	56M	BM	T3N0M0	No	No	Down	N/D
10	187	83M	BM	T2N1M0	Chronic Smok	No	Up	Up
11	188	33M	Tongue, anterior 2/3	T4N0M0	No	Yes	Up	Up
12	194	48F	GBS	T4N2bM0	Chronic Tob chewing	No	Down	Down
13	197	59M	BM	T2N0M0	Smok and Alco	Yes	Up	Up
14	199	50M	BM	T4N2M0	Tob chewing	No	Down	No change
15	204	70M	BM	T3N1M0	Chronic Tob chewing	No	Up	N/D
16	206	44M	Tongue-anterior 2/3	T3N2bM0	Chronic Tob chewing	No	Up	Up
17	211	65M	BM	T2N0M0	No	No	Up	N/D
18	213	37M	Tongue, anterior 2/3	T3N0M0	No	No	Up	Up
19	215	65F	BM	T2N1M0	No	No	No change	N/D
20	217	45F	BM	T3N1M0	Chronic Bet chewing	No	Up	N/D
21	219	68M	BM	T3N2bM0	Chronic Tob chewing	No	Up	Up
22	227	227	BM	T1N1M0	Tob chewing and Smok	No	No change	N/D
23	228	54F	BM	T2N0M0	Tob chewing	No	Down	N/D
24	233	50F	BM	T2N0M0	Tob chewing	No	Up	Up
25	234	58M	Tongue, anterior 2/3	T2N0M0	Bet and Betel quid chewing	No	Up	N/D

Abbreviations: Pt#, patient number; M, male; F, female; BM, buccal mucosa; GBS, gingivo-buccal sulcus; TNM, tumor node metastasis; Tob, tobacco; Alco, alcohol; Bet, betelnut; Smok, smoking; yes, amplified; no, not amplified; up, upregulated; down, downregulated; and, N/D, experiment not done.

Supplementary Table S2. Details of primers used in qPCR and qRT-PCR.

Gene	Sequence (5´ to 3´)	Amplicon size (bp)	Annealing temperature (^{0}C)	Reference
ESRRA	F: CGAGAGGAGTATGTTCTAC<br>R: TGCAGAGCTTCTCGCAGCT	110	59	
GAPDH	F:GAAGGGTGAAGGTCGGAGTC<br>R:GAAGATGGTGATGGGATTTC	226	60	
WNT11	F: GGACACAAGACAGGCAGTGCA<br>R:CCTCACTTGCAGACATAGCG	190	60	
OPN	F:AGACCTGACATCCAGTACCCTG<br>R:GTGGGTTTCAGCACTCTGGT	189	60	
WT1	F:TCTTGTACGGTCGGCATCTGAG<br>R:GTTCACAGTCCTTGAAGTCACAC	158	58	
STIL	F:CTTAAGCAACTAAGAAGCCTTGGAG<br>R: GCCTCCATGCTCAAATCCACACCA	197	49	1
miR-125a	RT6-miR-125a:<br>TGTCAGGCAACCGTATTCACCGTGAGTGG<br>TTCACAG<br>Short-miR-125a:<br>CGTCAGATGTCCGAGTAGAGGGGGAACG<br>GCGTCCCTGAGACCCTTTAACC<br><br>MP-F: TGTCAGGCAACCGTATTCACC<br>MP-R: CGTCAGATGTCCGAGTAGAGG	86	56	2, 3
5S RNA	F:GCCCGATCTCGTCTGATCT<br>R:AGCCTACAGCACCCGGTATT	110	60	3

Abbreviations: F, forward primer; and R, reverse primer.

Supplementary Table S3. Details of primers used in cloning.

Construct	Cloning vector	Sequence (5´ to 3´)	Amplicon size (bp)	Annealing temperature (^{0}C)
p*ESRRA*	pcDNA3.1(+)	F: TAGCAAGCTTATGTCCAGCCAGGTGGTGGGCATTG<br>*Hind* III<br>R:TGACGAATTCGAGGAGAGGGCGTGGGGCCTGTTC<br>*Eco R*I	1432	52

Abbreviations: F, forward primer; and R, reverse primer.

References

1. Kumar, A., Girimaji, S. C., Duvvari, M. R. & Blanton, S. H. Mutations in *STIL*, encoding a pericentriolar and centrosomal protein, cause primary microcephaly. Am. J. Hum. Genet. **84**, 286-290 (2009).
2. Tiwari, A., Shivananda, S., Gopinath, K. S. & Kumar, A. microRNA-125a reduces proliferation and invasion of oral squamous cell carcinoma cells by targeting estrogen-related receptoralpha: implications for cancer therapeutics. J. Biol. Chem. **289**, 32276-32290 (2014).
3. Sharbati-Tehrani, S., Kutz-Lohroff, B., Bergbauer, R., Scholven, J. & Einspanier, R. miR-Q: a novel quantitative RT-PCR approach for the expression profiling of small RNA molecules such as miRNAs in a complex sample. BMC Mol. Biol. **9**, 34 (2008).
